# Comparison of upfront versus deferred cytoreductive nephrectomy in patients with metastatic renal cell carcinoma receiving systemic therapy: a systematic review and meta-analysis

**DOI:** 10.1097/JS9.0000000000000591

**Published:** 2023-07-14

**Authors:** Kun-peng Li, Miao He, Shun Wan, Si-yu Chen, Chen-yang Wang, Xiao-ran Li, Li Yang

**Affiliations:** a Department of Urology; b Laboratory Medicine Center, The Second Hospital of Lanzhou University, Lanzhou, China

**Keywords:** deferred cytoreductive nephrectomy, immunotherapy, outcomes, targeted therapy, upfront cytoreductive nephrectomy

## Abstract

**Background:**

This study aimed to conduct a pooled analysis to compare the outcomes of patients with metastatic renal cell carcinoma who received presurgical systemic therapy [(ST); including immunotherapy and/or targeted therapy] followed by cytoreductive nephrectomy (CN) [(deferred CN; (dCN)] with those who underwent upfront CN (uCN) followed by ST.

**Methods:**

The present study followed the Preferred Reporting Items for Systematic Reviews and Meta-Analyses (PRISMA) statement. A comprehensive search was conducted in PubMed, Embase, Web of Science, Scopus, and the Cochrane Library database to identify eligible comparative studies up to April 2023. To evaluate their relevance, pooled hazard ratio with 95% CIs were calculated.

**Results:**

A total of 3157 patients were included in nine studies. The dCN group was observed to be correlated with superior overall survival (OS) compared to the uCN group (hazard ratio =0.71, 95% CI 0.57–0.89, *P*=0.003). Moreover, the authors conducted subgroup analyses according to the type of ST, sample size, sex, age, and risk score, and observed similar outcomes for OS across most subgroups.

**Conclusions:**

The results of this study demonstrated that dCN may be associated with improved OS compared to uCN in patients with metastatic renal cell carcinoma receiving ST. However, no significant differences were found between the uCN and dCN groups in the immunotherapy-based combinations subgroup. Further research is needed to confirm these results.

## Introduction

Highlights

- Nine high-quality published studies were included.
- All the studies were published between 2019 and 2022.
- The deferred cytoreductive nephrectomy was observed to be correlated with superior overall survival compared to the upfront cytoreductive nephrectomy.
- We observed similar outcomes for overall survival across most subgroups.

In two randomized clinical trials (RCTs) conducted in the early twenty-first century, a survival benefit was demonstrated in patients with metastatic renal cell carcinoma (mRCC) treated with cytoreductive nephrectomy (CN) in combination with interferon alpha therapy compared to those receiving interferon alpha therapy alone^1,2^. In 2004, targeted therapy was approved, rapidly supplanting cytokine-based therapy and becoming the standard of care over the subsequent decade^3^. Nowadays, immune checkpoint inhibitor therapy (immunotherapy) either alone or in combination with targeted therapy has demonstrated superior efficacy and has been adopted as the primary systemic therapy (ST)^4–6^. The developments of more effective ST for mRCC have called into question the role of CN. Recently, the results from a prospective RCT, CARMENA^7^, suggested that patients with mRCC who received sunitinib alone had comparable overall survival (OS) to those who underwent CN followed by sunitinib. Nevertheless, the recruitment delay and unbalanced proportion of patients with poor risk diseases observed in the CARMENA study population have raised some concerns regarding the universality and availability of this trial.

Esagian *et al.* ^8^ conducted a meta-analysis comprising 14 studies, which demonstrated that CN could be of benefit to selected patients undergoing targeted therapy. Furthermore, two retrospective studies provided preliminary evidence regarding the oncological role of CN in the immunotherapy era, including the timing and safety of its administration in relation to immunotherapy^9,10^. Nevertheless, the optimal time for CN has yet to be conclusively determined. Recent research has evaluated presurgical ST in advance of planned CN in single-arm trials^11–13^. The latest NCCN guidelines suggest initial ST as the preferred course of action for patients with intermediate and poor risk mRCC. However, due to the rapid developments in mRCC management, further research is needed to define the optimal timing for CN^14^. Moreover, the results remain controversial due to the relatively small sample size from different clinical centers, thus limiting our ability to draw reliable conclusions.

In order to aid clinicians in their decision-making process, we conducted a systematic review and meta-analysis to compare the survival outcomes of upfront CN (uCN) with deferred CN (dCN) for the treatment of patients with mRCC receiving ST.

## Methods

This study was conducted in accordance with the Preferred Reporting Items for Systematic Reviews and Meta-Analyses (PRISMA) 2020 guidelines^15,16^, Supplemental Digital Content 1, http://links.lww.com/JS9/A763. The quality assessment was performed according to Assessing the Methodological Quality of Systematic Reviews (AMSTAR) 2^17^, Supplemental Digital Content 2, http://links.lww.com/JS9/A764, and was registered in PROSPERO.

### Literature search strategy, study selection, and data collection

We conducted a systematic search of the Science, PubMed, Web of Science, Scopus, and the Cochrane Library database to identify published studies up to April 2023. The search terms were as follows: ((Metastasis Renal cell carcinoma OR metastasis kidney carcinoma OR metastasis renal cell cancer) AND (Cytoreductive nephrectomy OR nephrectomy OR radical nephrectomy) AND (Upfront OR immediate OR before) AND (Deferred OR after) AND (Immunotherapy OR immune checkpoint inhibitor OR PD-1 inhibitor OR anti-PD-1 inhibitor) AND (Targeted therapy OR tyrosine kinase inhibitor OR systemic therapy)). In addition, we conducted a manual search of relevant references and abstracts to ensure no pertinent results were omitted from our search scope.

We employed the PICOS approach to define the inclusion criteria: P (patients): all the patients were diagnosed with mRCC; I (intervention): patients underwent CN followed by ST (including immunotherapy and/or targeted therapy; uCN); C (comparator): patients received presurgical ST followed by CN (dCN); O (outcome): survival outcomes; S (study type): prospective studies, retrospective studies, or RCTs. Exclusion criteria include: duplicate and noncomparative studies; the type of reviews, editorial comments, case reports, meeting abstracts, and unpublished studies, and studies with insufficient or unavailable data for analysis.

Two reviewers comprehensively assessed all eligible literature. For each study, the following information was extracted: first author, year of publication, country, and duration of study; sample size, age, sex, clear cell carcinoma, and follow-up period; metastatic sites, International Metastatic Renal Cell Carcinoma Database Consortium (IMDC) or Memorial Sloan-Kettering Cancer Center (MSKCC) risk score, number of sites of metastasis, and type of ST; OS. Any differences and disagreements were reconciled by a third evaluator.

In this meta-analysis, we used the Cochrane Collaboration’s tool to assess the quality of the enrolled RCTs^18^, while the risk of bias in nonrandomized studies of interventions (ROBINS-I) was used to evaluate the non-RCTs^19^. Two reviewers carried out independent quality assessments of the included literature, and any disagreements were resolved through discussion.

### Statistical analysis

This study was conducted using the Cochrane Collaborative RevMan5.4 software to analyze the data, with a pooled hazard ratio (HR) being used to assess the survival outcomes and 95% CIs presenting the results. The heterogeneity of each indicator was determined with the *I* ^2^ test^20^, and statistical significance was set at *P* < 0.05. Additionally, the funnel plot, Egger’s test, and Begg’s test were used to assess the publication bias.

### Subgroup analysis

The subgroup analysis was conducted based on the type of ST, age, sex, sample size, and risk score.

## Results

### Baseline characteristics

**Figure 1:**
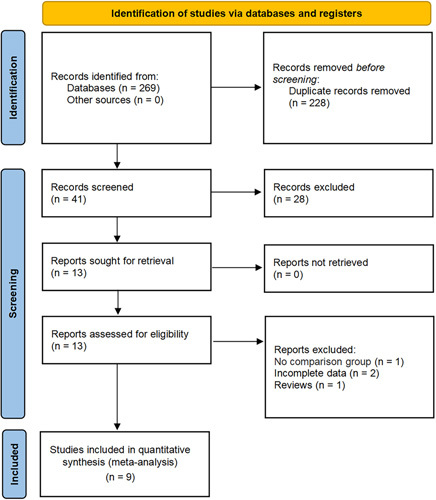
Preferred Reporting Items for Systematic Reviews and Meta-Analyses flow diagram for the systematic review.

A total of 269 studies were initially identified through electronic search, of which 13 were retained after the removal of duplicates. Through a rigorous reading and screening of the abstracts and full text, nine studies (one RCTs and eight non-RCTs) involving a total of 3157 patients were included in the meta-analysis (2519 uCN vs. 638 dCN) (Fig. 1)^21–29^. Eight non-RCTs that adopted a retrospective comparison design were conducted across multiple institutions. Three of these used immunotherapy^21–23^, four made use of targeted therapy^25,26,28,29^, and the remaining employed a combination of immunotherapy-based combinations^24,27^. The studies were conducted in a variety of regions, including the USA, Japan, the Netherlands, Germany, the UK, Italy, and Canada, with a follow-up period ranging from 1 to 3 years. One of the studies included in the analysis reported the median OS of two groups, with figures of 26.1 and 36.5 months in the uCN and dCN groups. Table 1 presents the characterizing particulars of the included studies, such as the country, age, sample size, sex, and follow-up duration. Table 2 summarizes the risk score, metastatic sites, number of sites of metastasis, and type of ST.

**Table 1:** The trials included in the systemic review

			Patients (*n*)		Age (years)		Male/Female		Race		Clear cell (*n*)		Time		Follow-up duration (months)	
References	Country	Study period	uCN	dCN	uCN	dCN	uCN	dCN	uCN	dCN	uCN	dCN	uCN	dCN	uCN	dCN
Gross^21^	USA	2000 and 2020	202	30	NA		155/47	25/5	White: 174; Black: 8; Asian:5; Other:14; Missing: 1	White: 24; Black:2; Asian:1; Other:2; Missing: 1	134	25	Time from mRCC to CN: 0.8 (0.2–1.4)	Time from mRCC to CN: 9.8 (5.1–22.5)	39.8 (17.1–66.6)	41.0 (24.7–73.3)
Yoshino^22^	Japan	September 2016 and July 2021	21	7	Median (IQR): 64.0 (53.5–69.5)	Median (IQR): 56.0 (47.0–64.0)	13/8	6/1	NA		18	5	dCN: patients with mRCC underwent ST 10 months before CN		12 (5–25)	19 (18–20)
Singla^23^	USA	2015–2016	197	24	Median (IQR): 56 (51–63)	Median (IQR): 65 (56–70)	150/47	17/7	White: 169; Black: 7; Hispanic: 13; Asian/Other: 8	White: 19; Black: 3; Hispanic: 2; Asian/Other: 0	NA		Time from mRCC to CN: 21 (7–38), days	Time from mRCC to CN: 123 (107–706), days.	Mean: 14.7	
Dragomir^24^	Canada	January 2011 and April 2020	383	73	Median (IQR): 61 (54–68)	Median (IQR): 62 (55–68)	279/104	54/29	NA		314	61	Time from mRCC to CN: 1 (0–2)	Time from mRCC to CN: 7 (5–11)	Followed up until the date of death, loss to follow-up, or the end of the study.	
Bruijn^25^	Multiple countries	2006 and 2016	149	189	mean (SD): 62.9 (10.8)	mean (SD): 61.6 (9.9)	103/46	142/47	NA		149	189	dCN: patients with mRCC underwent ST 12–18 weeks before CN		NA	
Bhindi^26^	Multiple countries	2006 and 2018	805	85	Median (IQR): 61 (54–68)		231/659		NA		757		dCN following ST: median 7.8 months from mRCC.		Median (IQR):25 (10–49)	
Ghatalia^27^	USA	2011 and 2020	605	142	Median (IQR): 62.3 (56–69)	Median (IQR): 63.2 (58–70)	418/187	101/41	White: 438; Black: 23; Hispanic: 6; Asian: 10; Other: 84; Missing:44	White: 101; Black: 8; Hispanic: 0; Asian: 2; Other: 21; Missing:10	NA		NA		NA	
Hatakeyama^28^	Japan	January 2008 and November 2019	107	39	Median (IQR): 62 (56–69)	Median (IQR): 67 (62–72)	79/28	22/17	NA		NA		dCN: patients with mRCC underwent ST 5.1 months before CN		NA	
Bex ^29^	Multiple countries	July 14, 2010 and March 24, 2016	50	49	Median (range): 60 (39–78)	Median (range): 58 (43–74)	41/9	39/10	NA		50	49	dCN: three cycles of sunitinib followed by CN		Median: 3.3 years.	

dCN, deferred cytoreductive nephrectomy; IQR, interquartile range; ST, systemic therapy; uCN, upfront cytoreductive nephrectomy.

**Table 2:** The trials included in the systemic review

	IMDC		Metastatic sites		Number of sites of metastasis		Type of immunotherapy	
References	uCN	dCN	uCN	dCN	uCN	dCN	uCN	dCN
Gross^21^	Favorable: 12; Intermediate:152; Poor: 35;	Favorable: 1; Intermediate:26; Poor: 3;	Lymph node: 53; Lung: 130; Liver: 18; Bone: 57; CNS: 3; Muscle: 4; Other kidney: 1; Others: 52	Lymph node: 7; Lung: 20; Liver: 4; Bone: 15; CNS: 2; Muscle: 2; Other kidney: 0; Others: 5	One: 101; Two or more: 88; Unknown: 13	One: 14; Two or more: 15; Unknown: 1	Ipilimumab and Nivolumab	
Yoshino^22^	Intermediate: 14; Poor: 7; Unknown: 0	Intermediate: 4; Poor: 5; Unknown: 1	Liver: 3; Bone: 7;	Liver: 1; Bone: 1;	Two or more: 13	Two or more: 5	Ipilimumab and Nivolumab	
Singla^23^	NA		Lung: 135; Liver: 15; Bone: 56; brain: 14	Lung: 12; Liver: 2; Bone: 17; brain: 0	Two or more: 47	Two or more: 8	Ipilimumab and Nivolumab	
Dragomir^24^	Favorable: 0; Intermediate: 253; Poor: 130;	Favorable: 0; Intermediate: 48; Poor: 25;	Lymph node: 122; Lung: 215; Liver: 36; Bone: 75; Adrenal gland: 41; Brain: 7	Lymph node: 28; Lung: 39; Liver: 9; Bone: 14; Adrenal gland: 10; Brain: 2	One: 252; 2 or more: 131;	One: 43; 2 or more: 30;	Targeted therapy and/or Immunotherapy	
Bruijn^25^	Intermediate: 131; Poor: 18	Intermediate: 144; Poor: 42	NA		One: 63; 2 or more: 86;	One: 49; 2 or more: 140;	Sunitinib, Pazopanib, and Sorafenib	
Bhindi^26^	Poor risk: 40%		Lymph node: 454; Lung: 659; Liver: 196; Bone: 338; Others: 267; Brain: 71		One: 249; 2 or more: 641		Sunitinib	
Ghatalia^27^	Intermediate: 90; Poor: 211; Poor/Intermediate: 304	Intermediate: 29; Poor: 47; Poor/Intermediate: 66	NA		NA		Targeted therapy and/or Immunotherapy	
Hatakeyama^28^	Intermediate: 63; Poor: 44	Intermediate: 15; Poor: 24	Lung: 74; Liver: 11; Bone: 38; Adrenal gland: 11; Brain:11	Lung: 23; Liver: 3; Bone: 14; Adrenal gland: 1; Brain:2	NA		Sunitinib, Sorafenib, Axitinib, and Pazopanib	
Bex^29^	Intermediate: 43; Poor: 7	Intermediate: 44; Poor: 5	Lung: 43; Liver: 8; Bone: 16; Adrenal gland: 10; Other soft tissue: 8;	Lung: 42; Liver: 7; Bone: 16; Adrenal gland: 14; Other soft tissue: 3;	One: 7; 2 or more: 43;	One: 3; 2 or more: 46;	Sunitinib	

dCN, deferred cytoreductive nephrectomy; IMDC, International Metastatic Renal Cell Carcinoma Database Consortium; MSKCC, Memorial Sloan-Kettering Cancer Center; uCN, upfront cytoreductive nephrectomy.

No statistically significant difference was observed between the two groups in terms of age (*P*=0.34), sex (*P*=0.76), clear cell histology (*P*=0.47), poor risk score (*P*=0.27), bone metastasis (*P*=0.20), lung metastasis (*P*=0.17), liver metastasis (*P*=0.58), brain metastasis (*P*=0.52), and adrenal gland metastasis (*P*=0.67) (Table 3).

**Table 3:** Comparison of baseline patient

Baseline characteristic	uCN versus dCN	Heterogeneity *I* ^2^ (%); *P*	*P*
Age WMD (95% CI)	−1.04 (−3.19–1.10)	72; 0.001	0.34
Female OR (95% CI)	0.96 (0.74–1.24)	11; 0.34	0.76
Clear cell OR (95% CI)	0.76 (0.35–1.62)	36; 0.21	0.47
Risk score (Poor); OR (95% CI)	0.79 (0.52–1.20)	53; 0.05	0.27
Metastatic sites: bone (95% CI)	0.66 (0.35–1.25)	68; 0.008	0.20
Metastatic sites: lung (95% CI)	1.26 (0.91–1.74)	0; 0.58	0.17
Metastatic sites: liver (95% CI)	0.87 (0.54–1.41)	0; 0.95	0.58
Metastatic sites: Brain (95% CI)	1.40 (0.50–3.95)	0; 0.43	0.52
Metastatic sites: Adrenal gland (95% CI)	0.85 (0.41–1.78)	32; 0.23	0.67

dCN, deferred cytoreductive nephrectomy; uCN, upfront cytoreductive nephrectomy.

### Assessment of quality

All the studies included in this analysis, published between 2019 and 2022, conducted comparative analysis. Of the eight non-RCTs included, six had a moderate risk of bias and two had a low risk of bias (Table S1, Supplemental Digital Content 3, http://links.lww.com/JS9/A765)^21,25^. Moreover, the one RCT was not double-blinded, resulting in an increased risk of bias, thereby classifying it as high risk (Fig.S1, Supplemental Digital Content 4, http://links.lww.com/JS9/A766)^29^.

### Outcome analysis

#### Overall survival

**Figure 2:**
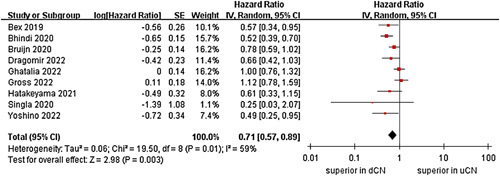
Forest plots of overall survival.

A meta-analysis of nine studies revealed that the OS of the dCN group was significantly more superior than that of the uCN group (HR=0.71, 95% CI 0.57–0.89, *P*=0.003), and the level of heterogeneity was moderate (*I* ^2^=59%; *P*=0.01) (Fig. 2)^21–29^.

#### Subgroup analyses

**Figure 3:**
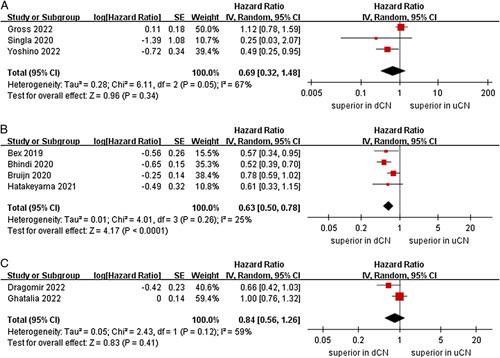
Forest plots of overall survival in subgroup analysis. (A) immunotherapy, (B) targeted therapy, (C) immunotherapy-based combinations.

**Figure 4:**
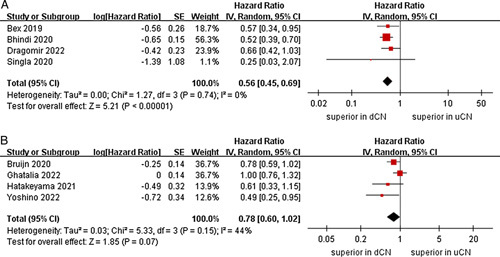
Forest plots of overall survival in subgroup analysis. (A) mean age less than 62 years, (B) mean aged greater than or equal to 62 years.

**Figure 5:**
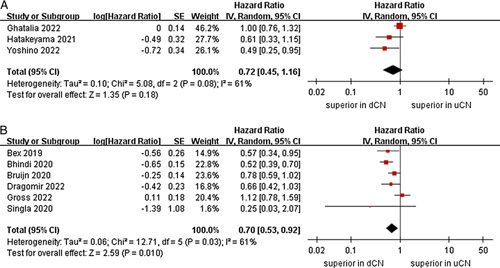
Forest plots of overall survival in subgroup analysis. (A) male proportion less than 70%, (B) male proportion greater than or equal to 70%.

**Figure 6:**
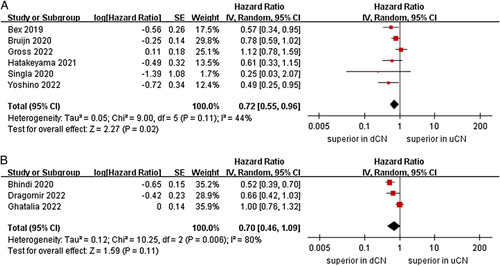
Forest plots of overall survival in subgroup analysis. (A) sample size less than 400, (B) sample size greater than or equal to 400.

**Figure 7:**
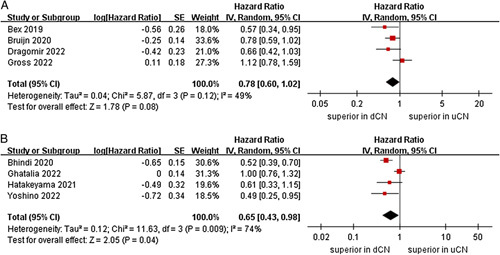
Forest plots of overall survival in subgroup analysis. (A) risk score (poor proportion) less than 35%, (B) risk score (poor proportion) greater than or equal to 35%.

We performed a stratified subgroup analysis for OS with a limited number of studies, based on the type of ST, age, sex, sample size, and risk score. According to stratification based on ST, in studies that involved immunotherapy, there is no statistical significance in the OS between dCN and uCN (three studies; *P*=0.34)^21–23^. In the targeted therapy subgroup, the dCN group was associated with a more favorable OS than the uCN group (HR=0.63, 95% CI 0.50–0.78, *P* < 0.0001; *I* ^2^=25%, *P*=0.26)^25,26,28,29^. In the immunotherapy-based combinations subgroup, no significant differences were found between the uCN and dCN groups (two studies; *P*=0.41) (Fig. 3)^24,27^. For subgroup of age, the meta-analysis demonstrated that the dCN group had a significantly lower risk of death compared to the uCN group in the mean age less than 62 years subgroup (HR=0.56, 95% CI 0.45–0.69, *P* < 0.00001; *I* ^2^=0%, *P*=0.74)^23–25,29^; conversely, no significant difference was observed in the mean aged greater than or equal to 62 years subgroup (*P*=0.07) (Fig. 4)^21,22,25,28^. Subgroup analyses stratified by sex, our results showed that no significant difference was observed between the two groups in the male proportion less than 70% subgroup (three studies; *P*=0.18)^22,27,28^. However, the dCN group was also observed to be correlated with superior OS than the uCN group in the male proportion greater than or equal to 70% subgroup (six studies; HR=0.70, 95% CI 0.53–0.92, *P*=0.01; *I* ^2^=61%, *P*=0.03) (Fig. 5)^21,23–26,29^. In the subgroup analysis with a sample size less than 400, the cumulative analysis indicated the dCN group exhibits a more favorable OS outcome compared to the uCN group (six studies; HR=0.72, 95% CI 0.55–0.96, *P*=0.02; *I* ^2^=44%, *P*=0.11)^21–23,25,28,29^. Furthermore, the pooled results revealed no difference between the two groups in the sample size greater than or equal to 400 subgroup (three studies; *P*=0.11) (Fig. 6)^24,26,27^. No significant difference in OS was observed between the two groups in the risk score (poor proportion) less than 35% subgroup (four studies; *P*=0.08). However, in the subgroup analysis of patients with a risk score (poor proportion) of greater than or equal to 35%, the dCN group demonstrated a significant correlation with superior OS compared to the uCN group (four studies; HR=0.65, 95% CI 0.43–0.98, *P*=0.04; *I* ^2^=74%, *P*=0.009) (Fig. 7)^22,26–28^.

### Sensitivity analysis

To further assess the source of heterogeneity, a sensitivity analysis was conducted by sequentially excluding one study at a time to observe the changes in heterogeneity of OS. The results revealed no remarkable changes in the heterogeneity of the studies, regardless of the study excluded, indicating the stability of the heterogeneity. The heterogeneity observed among the studies may be attributed to various factors, including the duration of follow-up, risk score, metastatic sites, systemic treatment modality, drug cycle, region, and racial characteristics.

### Publication bias

We evaluated the potential for publication bias using the funnel plot, Egger’s test, and Begg’s test. The analysis revealed no significant evidence of publication bias based on the results obtained from the funnel plot, Egger’s test (*P*=0.245), and Begg’s test (*P*=0.251) (Fig. S2, Supplemental Digital Content 5, http://links.lww.com/JS9/A767).

## Discussion

This is the first systematic review and meta-analysis to compare the survival outcomes of uCN with dCN for the treatment of patients with mRCC receiving ST. Furthermore, the findings from this analysis merit further discussion.

Given the emergence of more effective systemic therapies for mRCC, the utility of CN has been called into question. However, an increasing body of evidence indicates that the combination of CN and ST may lead to improved outcomes in mRCC. Roussel *et al.* ^30^ demonstrated that patients undergoing upfront ST in conjunction with CN may have a more favorable survival outcomes compared to those who only received ST. Gross *et al.* ^21^ conducted a study, which demonstrated that CN is an independent prognosticator for OS in patients with mRCC who were treated with immunotherapy, and their data suggest that, for selected patients with mRCC, CN may be a viable treatment option when combined with immunotherapy. CN has been demonstrated to be effective in decreasing the overall tumor burden, thereby prolonging the period prior to the progression of tumors to a life-threatening state^31^. Moreover, the efficacy of combining CN with ST has been further demonstrated in metastatic tumors such as lung cancer and melanoma, lending support to its use in mRCC treatment^32,33^.

However, the timing of CN for patients with mRCC remains a topic of debate in both the literature and the clinic. The NCCN guidelines acknowledge the need for further research to resolve this issue. The concept of controlling the disease in patients who need systemic treatment with drug therapy instead of surgery first may be applied universally. Ghanem *et al.* ^34^ reported that the cumulative reduction of target lesions was more significant in the cohort that underwent delayed CN treatment than those who had immediate CN therapy. Some studies have suggested that dCN may be beneficial as ST can decrease tumor size and neovascularisation, thereby facilitating resection. Moreover, nephrectomy may alleviate symptoms, such as pain or hematuria, which can have a negative impact on quality of life^35–37^. The prospective CARMENA trial conducted by Méjean *et al.* ^7^ showed that secondary nephrectomy was associated with improved OS compared to no nephrectomy in the sunitinib alone arm. Hence, these findings may support the potential of dCN in some mRCC patients. At present, there is little evidence from prospective data to support the concept of deferred CN when combined with ST. However, RCTs that investigate the efficacy of this practice have begun to recruit participants (NCT03977571 and NCT04510597).

Bruijn *et al.* ^38^ conducted a RCT to assess the safety of performing surgery after targeted therapy. The results of the trial demonstrated that surgery following targeted therapy is secure. Administering an initial course of ST might act as an indicator to determine which mRCC patients have an aggressive biology. In other words, presurgical targeted therapy appeared to identify patients with rapid disease progression, most likely due to their inherent resistance to the treatment. This could be seen as a kind of litmus test for ascertaining poor prognoses^11,12^. For instance, Powles *et al.* ^11^ conducted a Phase II trial involving 66 participants who were administered ST treatment prior to undergoing planned CN, from which 27% encountered progression of the disease either prior to or at the time of the scheduled surgery. Furthermore, it has been suggested that a major issue associated with the uCN approach is the possibility of not being able to receive subsequent ST for up to 15–30% of patients, mainly due to postoperative complications or swift disease progress^39^. Similarly, in the prospective CARMENA trial, 18% of patients in the uCN group did not receive postoperative sunitinib treatment^7^. However, there are no relevant reports on incidence rates in the studies we included. Two studies reported that if a patient in the dCN group exhibited systemic progressive disease, nephrectomy was canceled. We need more studies to verify the advantages of dCN compared to uCN.

Patient selection is a key factor to be taken into account when assessing the efficacy of CN^40^. Bakouny *et al.* ^9^ suggested that patients without unfavorable bone, liver or lung metastases, a favorable IMDC risk score, and good physical condition may gain substantial benefit from CN. Among the included studies, four had a risk score (poor proportion) higher than 35%, while the other four had a risk score (poor proportion) lower than 35%. Although no statistically significant difference in OS was observed between the two groups in the risk score (poor proportion) less than 35% subgroup (four studies; *P*=0.08), the subgroup analysis of patients with a risk score (poor proportion) greater than or equal to 35% revealed a noteworthy finding. The dCN group exhibited a significant correlation with superior OS compared to the uCN group (four studies; HR=0.65, 95% CI 0.43–0.98, *P*=0.04; *I* ^2^=74%, *P*=0.009). This outcome could be attributed to the paucity of literature included. Furthermore, in the one included study (26), no significant OS difference between all patients receiving uCN versus dCN. On the contrary, in the IMDC (intermediate) subgroup, a significant OS benefit (*P*<0.001) was noted in the dCN compared to the uCN. Therefore, further studies should be conducted to determine the risk scores of patients who could gain advantages from dCN and to evaluate the results of such treatment.

The timing of dCN also remains an issue of debate. Different timings of dCN is reported in the included literature. In the targeted therapy, the SURTIME trial reported that dCN was performed three cycles after the commencement of the initial sunitinib treatment reported^29^. In addition, Bhindi *et al.* ^26^ reported that the median interval for performing dCN after ST administration was 7.8 months from the time of diagnosis. Bruijn *et al.* ^25^ showed that ST was conducted in the dCN group 12–18 weeks prior to CN. Furthermore, in the immunotherapy, Singla *et al.* ^23^ showed that the median time between diagnosis and the beginning of dCN treatment following immunotherapy was 123 days. Additionally, Yoshino *et al.* ^22^ showed that the implementation of dCN occurred 10 months after commencing the course of nivolumab and ipilimumab. The collective results indicated that, so long as patients could maintain sufficient control of the disease for a period of at least 3–10 months after the commencement of ST, dCN did not appear to have a deleterious effect on prognosis. However, further rigorous research is necessary to assess whether the timing of dCN could bring survival benefits to patients with mRCC.

It is necessary to acknowledge certain limitations of our study. First, most of the included studies were retrospective with intermediate quality, thereby potentially incurring distribution and blindness bias. Second, the patients included were from large centers and may not accurately reflect the general population. Third, the absence of sufficient data within the included studies posed a limitation on our ability to perform a comprehensive pooled analysis for assessing other survival outcomes, such as progression-free survival. Fourth, although the included studies included different racial groups (e.g. White, Black, and Asian), the limited literature available barred us from conducting subgroup analysis, which may result in some heterogeneity. Lastly, only one of the studies included in the analysis reported the median OS of the two groups. However, it is important to provide actual survival data in terms of weeks or months when discussing treatment options with patients diagnosed with mRCC.

## Conclusions

The results of this study indicated that dCN may be correlated with superior OS than uCN in patients with mRCC who received ST. However, no significant differences were found between the uCN and dCN groups in the immunotherapy-based combinations subgroup. In order to confirm these findings, further studies with larger sample sizes, longer follow-up periods, and RCTs are needed.

## Ethical approval

Not applicable.

## Sources of funding

This study was supported by the National Natural Science Foundation of China (No. 82160146); Cuiying Scientific and Technological Innovation Program of Lanzhou University Second Hospital (Grant numbers CY2021-MS-A12 and CY2020- MS08 and 2020QN-09); Natural Science Foundation of Gansu Province of China (Grant numbers 21JR1RA151).

## Author contribution

K.P.L., M.H., and S.W.: conceived and designed the experiments; K.P.L., M.H., S.W., S.Y.C., X.R.L., and L.Y.: analyzed the data; K.P.L., M.H., S.Y.C., and C.Y.W.: contributed reagents/materials/analysis; K.P.L., M.H., S.W., S.Y.C., X.R.L., and L.Y.: wrote the manuscript. All authors have read and approved the final manuscript.

## Conflicts of interest disclosure

All the authors have nothing to declare.

## Research registration unique identifying number (UIN)

1. Name of the registry: PROSPERO database.
2. Unique Identifying number or registration ID: CRD42023396026.
3. Hyperlink to your specific registration (must be publicly accessible and will be checked): https://www.crd.york.ac.uk/prospero/display_record.php?RecordID=396026

## Guarantor

Li Yang.

## Data availability statement

All data generated and analyzed during this study are included in this published article. The data presented in the article may be requested by consulting the correspondence author.

## Supplementary Material

SUPPLEMENTARY MATERIAL
