# Supplementary material for: Comparison of upfront versus deferred cytoreductive nephrectomy in patients with metastatic renal cell carcinoma receiving systemic therapy: a systematic review and meta-analysis
Source: Int J Surg. 2023 Jul 14;109(10):3178–88. doi: 10.1097/JS9.0000000000000591 (PMC10583944; doi:10.1097/JS9.0000000000000591)
Supplement: SUPPLEMENTARY MATERIAL [file js9-109-3178-s003.docx]

| **Table S1 The risk of bias (Non-RCTs)-ROBINS-I** | | | | | | | | |  |
| --- | --- | --- | --- | --- | --- | --- | --- | --- | --- |
| Bias domain | Gross | Yoshino | Singla | Dragomir | Bruijn | Bhindi | Ghatalia | Hatakeyama |  |
|  |  |  |  |  |  |  |  |  |  |
| Bias due to confounding | Moderate | Moderate | Moderate | Moderate | Moderate | Moderate | Moderate | Moderate |  |
|  |  |  |  |  |  |  |  |  |  |
| Bias in selection of participants into the study | Low | Low | Low | Low | Low | Low | Low | Low |  |
|  |  |  |  |  |  |  |  |  |  |
| Bias in classification of interventions | Low | Low | Low | Low | Low | Low | Low | Low |  |
|  |  |  |  |  |  |  |  |  |  |
| Bias due to deviations from intended interventions | Low | Moderate | Low | Low | Moderate | Low | Low | Low |  |
|  |  |  |  |  |  |  |  |  |  |
| Bias due to missing data | Moderate | Moderate | Moderate | Moderate | Moderate | Moderate | Moderate | Moderate |  |
|  |  |  |  |  |  |  |  |  |  |
| Bias in measurement of outcomes | Low | Moderate | Moderate | Low | Low | Moderate | Moderate | Low |  |
|  |  |  |  |  |  |  |  |  |  |
| Bias in selection of the reported result | Low | Low | Moderate | Moderate | Low | Moderate | Moderate | Moderate |  |
|  |  |  |  |  |  |  |  |  |  |
| Overall bias | Low | Moderate | Moderate | Moderate | Low | Moderate | Moderate | Moderate |  |
|  |  |  |  |  |  |  |  |  |  |
