# Supplementary figures and images for: Comparison of upfront versus deferred cytoreductive nephrectomy in patients with metastatic renal cell carcinoma receiving systemic therapy: a systematic review and meta-analysis
Source: Int J Surg. 2023 Jul 14;109(10):3178–88. doi: 10.1097/JS9.0000000000000591 (PMC10583944; doi:10.1097/JS9.0000000000000591)

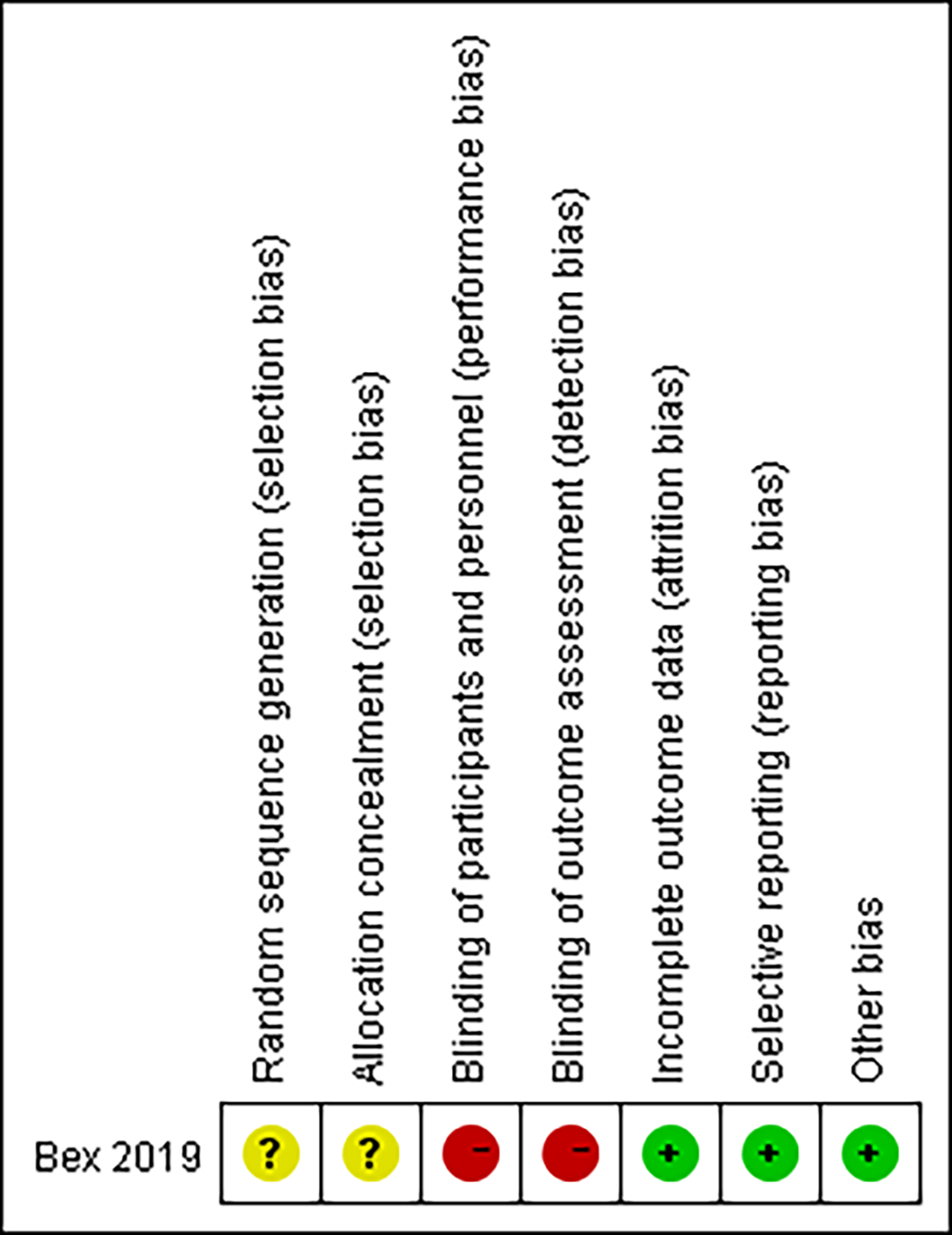

Supplement: SUPPLEMENTARY MATERIAL [file js9-109-3178-s004.tif]

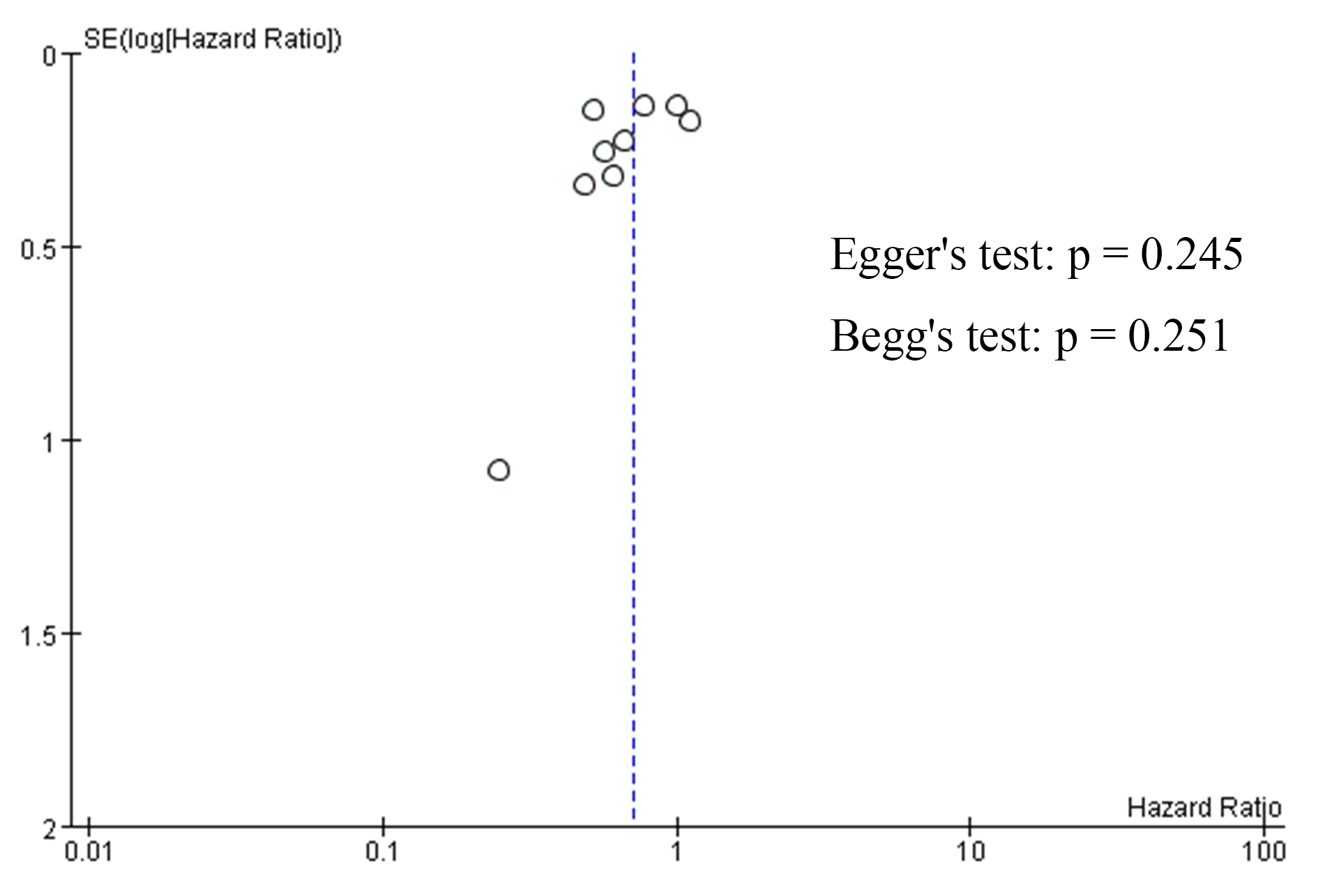

Supplement: SUPPLEMENTARY MATERIAL [file js9-109-3178-s005.tif]
